# Plasma cell survival in the absence of B cell memory

**DOI:** 10.1038/s41467-017-01901-w

**Published:** 2017-11-24

**Authors:** Erika Hammarlund, Archana Thomas, Ian J. Amanna, Lindsay A. Holden, Ov D. Slayden, Byung Park, Lina Gao, Mark K. Slifka

**Affiliations:** 1 Division of Neuroscience, Oregon National Primate Research Center, Oregon Health & Science University, 505 NW 185th Avenue, Beaverton, OR 97006 USA; 2 Najít Technologies, Inc, 505 NW 185th Avenue, Beaverton, OR 97006 USA; 3 Department of Biology, Portland State University, 1719 SW 10th Avenue, Portland, OR 97201 USA; 4 Division of Reproductive Sciences, Oregon National Primate Research Center, Oregon Health & Science University, 505 NW 185th Avenue, Beaverton, OR 97006 USA; 5 Biostatistics Shared Resource, Knight Cancer Institute, 3181 SW Sam Jackson Park Rd., Portland, OR 97239 USA

## Abstract

Pre-existing serum antibodies play an important role in vaccine-mediated protection against infection but the underlying mechanisms of immune memory are unclear. Clinical studies indicate that antigen-specific antibody responses can be maintained for many years, leading to theories that reactivation/differentiation of memory B cells into plasma cells is required to sustain long-term antibody production. Here, we present a decade-long study in which we demonstrate site-specific survival of bone marrow-derived plasma cells and durable antibody responses to multiple virus and vaccine antigens in rhesus macaques for years after sustained memory B cell depletion. Moreover, BrdU^+^ cells with plasma cell morphology can be detected for 10 years after vaccination/BrdU administration, indicating that plasma cells may persist for a prolonged period of time in the absence of cell division. On the basis of these results, long-lived plasma cells represent a key cell population responsible for long-term antibody production and serological memory.

## Abstract (web summary)

The long-term maintenance of antibody-secreting plasma cells and the requirement for memory B cells are unclear. Here, the authors show that plasma cells and the antibodies secreted are long-lived and maintained over a decade in the absence of memory B cells in non-human primates.

## Introduction

The question of plasma cell longevity and its role in maintaining serum antibody levels has sparked considerable debate over the past 50 years. Studies from the 1960's noted that plasma cells had a half-life of only a few days at the early stages of an immune response^1–4^, whereas later studies found that plasma cells could survive for weeks/months^5–7^ or potentially even longer^8^. Our initial studies in mice demonstrated that long-lived plasma cells could survive in the absence of memory B cells^9^ and similar observations have been demonstrated in a number of animal models^10–12^. Although plasma cells were detected up to a year or more after irradiation-induced memory B cell depletion in mice^9^, antigen-specific serum antibody declined compared to those of untreated controls. Consequently, there has been a resurgence of theories regarding the potential importance of cell proliferation^13,14^, persisting antigen^15,16^ or non-specific activation of memory B cells^16–18^ to sustain plasma cell numbers and antibody levels over the course of a human lifespan. To investigate this question in more detail, here we show naturally acquired and vaccine-mediated immune responses in rhesus macaques that persist up to a decade after immunization and demonstrate the existence of long-lived plasma cells that can independently maintain serum antibody levels for many years in the absence of memory B cells.

## Results

### Antibody decay rates pre and post memory B cell depletion

**Fig. 1:**
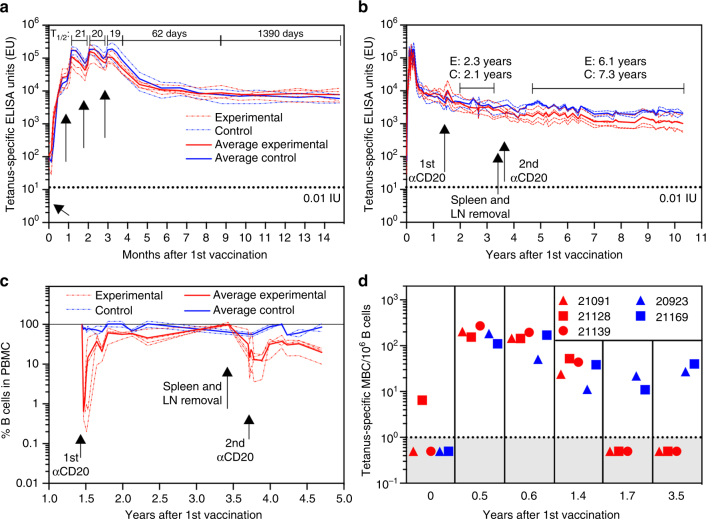
Tetanus-specific antibody responses following memory B cell depletion. Six Rhesus macaques received 4 intramuscular doses of Tripedia^®^ vaccine (arrows, panel **a**) and tetanus-specific serum antibody responses were monitored closely for 15 months **a** to 10 years **b**. CD20^+^ B cells were depleted from 4 experimental animals (Experimental; E) at the indicated time points by administration of anti-CD20/Rituximab (**b**, **c**) and these animals also underwent splenectomy and surgical removal of draining lymph nodes (LN) at 3.5 years after primary vaccination. Two control animals (Control; C) were monitored in parallel throughout the experiment to compare tetanus-specific antibody levels and memory B cell frequencies. Efficiency of B cell depletion was determined by staining PBMC for CD22^+^ B cells **c** and tetanus-specific memory B cells were directly measured by flow cytometry either before or after memory B cell depletion performed at 1.5 years after vaccination **d**. The dashed line in **a** and **b** represents the tetanus-specific ELISA titer coinciding with 0.01 IU/ml calibrated based on the international serum standard, Tetanus Immunoglobulin TE-3. The dashed line in **d** indicates the limit of detection. Further details describing the statistical model for determining antibody decay rates and half-life estimates can be found in the Methods (Eqs. 1 and 2)

Rhesus macaques were immunized against tetanus using a commercially available vaccine (DTaP, Tripedia^®^). This represents a common childhood vaccine antigen and the tools for measuring antibody levels and memory B cell responses to tetanus are well established^19,20^. The animals received four intramuscular doses of vaccine at one-month intervals and we examined the magnitude and durability of tetanus-specific immune responses for ~10 years (*n* = 6 rhesus macaques and > 550 serum samples, Fig. 1). Antibody decay rates were measured during the first month after each booster vaccination and found to have an antibody half-life of 19–21 days, similar to the decay rate of IgG molecules themselves^21–24^. This indicates that most of the antibody-secreting cells (ASC) induced early after vaccination are very short-lived (Fig. 1a). From 1 to 6 months after the last vaccination, there was a clear biphasic decay curve in which the estimated antibody half-life increased to 62 days. This is in contrast to the more stable tetanus-specific antibody half-life of 1390 days observed from 6 to 12 months after final vaccination (i.e., 9–15 months after primary vaccination).

At 1.5 years after primary vaccination, 4 experimental animals had CD20^+^ memory B cells depleted by the intravenous administration of 3 weekly doses of anti-CD20 antibody (Rituximab®, 20 mg/kg) and 2 control animals did not receive anti-CD20 depletion but blood samples continued to be drawn on a similar schedule (Fig. 1b). Early analysis of antibody decay rates after memory B cell depletion indicated an average tetanus-specific antibody half-life of 2.3 years and 2.1 years for the experimental and control groups, respectively and these were not significantly different (*P* = 0.80, Mann–Whitney test). However, studies in humans indicate that anti-CD20 depletion may not be as effective at removing B-cells from lymphoid tissues as it is for depleting B cells from the circulation^25–31^, an outcome that might be related to the degree of inflammation at the time of administration^32^. To eliminate this potential caveat, the spleen and inguinal lymph nodes (i.e., the draining lymph nodes after vaccination in the quadriceps muscle) were surgically removed from the experimental animals at ~3.5 years after primary vaccination and intravenous anti-CD20 depletion was repeated (three doses at weekly intervals, 20 mg/kg). Analysis of B cell frequencies in the peripheral blood indicated that the first round of anti-CD20 depletion removed > 99% of B cells from circulation and the second round of anti-CD20 depletion reduced circulating B cell numbers by ~ 85%. Recovery of peripheral B cell numbers only reached an average of ~ 20% of the pre-depletion levels at one year after the second treatment (Fig. 1c). In addition to monitoring total B cell depletion, we also measured antigen-specific memory B cell numbers by flow cytometry^19^ (Fig. 1d). Tetanus-specific memory B cell frequencies increased from < 10/10^6^ B cells prior to vaccination to an average of 185 ± 60 (standard deviation, *n* = 5) memory B cells/10^6^ B cells at 0.5 years after the first vaccination (i.e., about 3 months after the last vaccination). At 1.4 years after primary vaccination, tetanus-specific memory B cell levels had declined to 34 ± 16 memory B cells/10^6^ B cells but remained detectable in the five animals that had sufficient PBMC for analysis. The first round of anti-CD20 depletion was performed at 1.5 years after vaccination and when tetanus-specific memory B cell frequencies were determined at 1.7 years (2.5 months after depletion), the tetanus-specific memory B cell population had dropped to below our limits of detection ( < 1/10^6^ B cells). In contrast, tetanus-specific memory B cells in the untreated control animals remained stable from 1.7 to 3.5 years after vaccination. This indicates that tetanus-specific B cell memory is long-lived in rhesus macaques but after anti-CD20 depletion and immune reconstitution of the general B-cell repertoire, tetanus-specific memory B-cells remained below detection when examined at 2.5 months or even 2 years later.

Following memory B cell depletion, the durability of tetanus-specific antibody responses was monitored longitudinally in comparison to non-depleted control animals from years 5 to 10 after primary vaccination (Fig. 1b). The tetanus-specific antibody half-life observed among the experimental memory B cell-depleted animals (*T*_*1/2*_ = 6.1 years, range; 4.7–8.2 years) was not significantly different from the control animals (*T*_*1/2*_ = 7.3 years, range: 5.2–12.2 years) (*P* = 0.80, Mann–Whitney test). At 10 years after primary vaccination, the memory B cell-depleted experimental group maintained an average anti-tetanus ELISA titer of 1015 ELISA Units (0.85 IU/ml) and based on a 6.1 year antibody half-life and a protective threshold of 0.01 IU/ml^33–36^, these vaccinated animals would be expected to remain protected against tetanus for nearly 50 years without requiring further vaccination—a time frame that exceeds the maximum lifespan of rhesus macaques (~ 40 years when raised in captivity^37^). Altogether, this indicates that after surgically removing potential B cell reservoirs from solid tissues such as the spleen and the draining lymph nodes, as well as all detectable tetanus-specific memory B cells from the circulation, tetanus-specific serum antibody titers continued to be maintained above the protective threshold for the lifespan of the immune host with decay rate kinetics that were indistinguishable from untreated controls.

### Durable antibody responses to multiple antigens

**Fig. 2:**
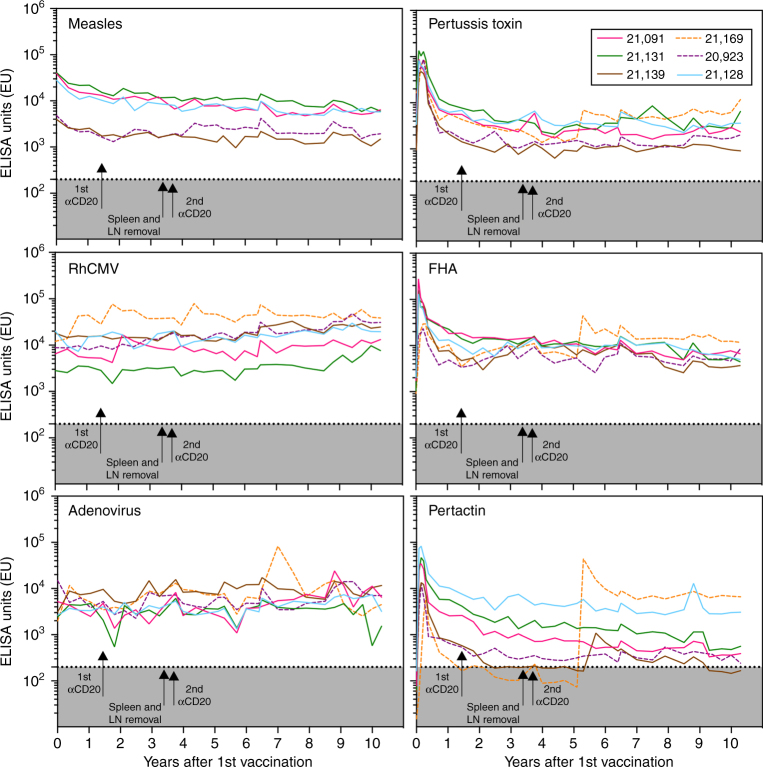
Longitudinal analysis of antibody responses to multiple antigens after vaccination or infection. Serum antibody titers were measured at the indicated time points for a paramyxovirus that is antigenically related to measles virus (Measles), rhesus cytomegalovirus (RhCMV), adenovirus, pertussis toxin, filamentous hemagglutinin (FHA), and pertactin. Arrows indicate the dates when anti-CD20 administration was performed or when splenectomy and draining lymph nodes (LN) were surgically removed. Control animals, Rh#20923 and Rh#21169, did not have anti-CD20 treatment or surgeries performed and are represented by dashed lines. The gray shaded region below the dotted line (<200 ELISA units) represents the points in which ELISA titers become equivocal or are below the limit of detection. Control animal Rh#21169 was seronegative for measles virus antigen

To determine if the durability of tetanus-specific antibody responses after memory B cell depletion were unique to this antigen or more broadly representative of immune responses to other types of vaccine antigens or infections, we measured antibody responses to *Bordetella pertussis* antigens (pertussis toxin, pertactin, filamentous hemagglutinin (FHA)), Rhesus cytomegalovirus (RhCMV), adenovirus, and a simian paramyxovirus that is antigenically related to measles virus (Measles) (Fig. 2 and Supplementary Fig. 1). Pertussis toxin, pertactin, and FHA are vaccine antigens included in the DTaP vaccine formulation and similar to tetanus, these antibody responses underwent rapid peaks and decay shortly after vaccination before reaching a plateau stage of more durable antibody responses by 2–3 years after the final vaccination. Both anti-CD20-depleted experimental animals and untreated control animals showed similar antibody responses to each of these pertussis antigens. Control animal #21169 appears to have been infected with *B. pertussis* at year 5 after vaccination because there was a spike in antibody titers to all three pertussis antigens. Experimental animal #21139 may have also been infected with *B. pertussis* since it showed a spike in pertactin-specific antibodies at year 5 after vaccination even though all of the animals were housed indoors from years 5 through 10 after vaccination. We speculate that they may have been exposed to infected animal husbandry staff during this period of time and this underscores the challenges associated with measuring long-term immunity to contagious pathogens.

RhCMV causes a persistent infection in macaques and as expected, we found that the antibody responses to this virus remained stable or showed a slow increase in titers over time. It is unclear how often animals are exposed/re-exposed to adenoviruses but we found that serological responses to this virus remained at high levels throughout the period of observation. In contrast, infection with a measles-like paramyxovirus provided the opportunity to measure immune responses to an infectious agent in the absence of known re-exposure. In 1999, a simian paramyxovirus outbreak occurred at the Oregon National Primate Research Center and infected a large number of animals as well as appearing to have infected up to 4 animal husbandry personnel^20^. The animals in this current study were born in 1999 and 5/6 of the animals seroconverted as indicated by the induction of antibodies that cross-react with measles antigen by ELISA (control animal #21169 remained seronegative). Paramyxoviruses typically cause acute viral infection and following the outbreak in 1999, no further outbreaks of the virus were identified prior to necropsy. Moreover, after the animals were brought indoors at year 5 of the study, there was little or no chance of possible re-exposure from other animals in the colony. This provided the opportunity to measure the duration of pre-existing antibody responses to a natural viral infection in the absence of re-exposure and in the absence of memory B cells after anti-CD20 depletion. The untreated control animal #20923 showed a measles-reactive antibody half-life of 10.6 years whereas the estimated antibody half-life among the anti-CD20 depleted animals was 13.0 years (#21092), 10.5 years (#21128), 6.5 years (#21131), and 310 years (#21139), respectively. Together, these results indicate that antibody responses to both vaccine and viral antigens can be maintained for many years by long-lived plasma cells without requiring continued replenishment by memory B cells.

### Localization of plasma cells to distinct bone marrow sites

**Fig. 3:**
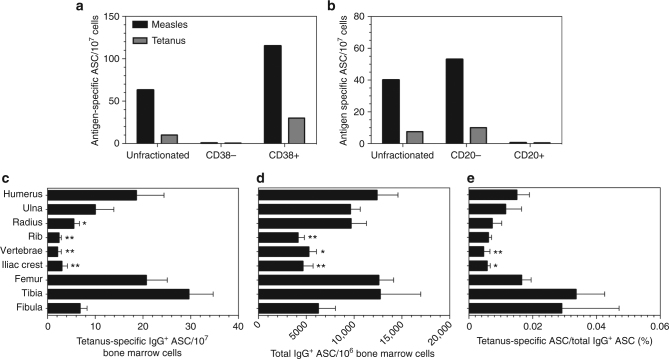
Antigen-specific and total IgG^+^ plasma cells in different bone marrow compartments. Quantitation of tetanus-specific, measles-specific, or total IgG-specific antibody-secreting cells (ASC) was determined directly ex vivo in 6-hour ELISPOT assays. **a**, **b** Samples of femoral bone marrow cells from a representative animal (Rh#21128) were assayed to determine the frequency of measles-specific and tetanus-specific ASC by ELISPOT from unfractionated bone marrow compared to the frequencies observed after MACS-based separation of **a** CD38^−^ vs. CD38^+^ cell populations or **b** CD20^−^ vs. CD20^+^ cell populations. The frequency of tetanus-specific ASC **c**, the frequency of total IgG-secreting ASC **d** and the frequency of tetanus-specific ASC as a percentage of total IgG-secreting ASC **e** was determined by ELISPOT analysis for each of the indicated bone marrow sites. The bars in **c**–**e** represent the mean ± S.E.M. Significant differences in plasma cell frequencies between the indicated bone marrow sites compared to the femur are indicated by Dunnett adjusted *P* values (**c**, **d**; mixed effect negative binomial regression, **e**; rank based mixed effect linear regression*, n* = 6, see also Supplementary Fig. 2). Symbols, *P < 0.05 and **P < 0.01

To further characterize the long-lived plasma cells identified in rhesus macaques, we determined their surface phenotype and localization within different bone marrow compartments (Fig. 3). In humans^38^, the long-lived bone marrow-derived plasma cell population is phenotypically defined as CD19^−^CD38^hi^CD138^+^. In our hands, CD19 expression on macaque B cells was relatively dim compared to human B cells^39^ and therefore we substituted CD20 as another common pan-B cell marker that is not expressed on human plasmablasts^40^ or long-lived human plasma cells^38^ but is highly expressed on macaque B cells^39,41^ and is suitable for magnetic activated cell sorting (MACS) experiments. At 10 years after vaccination, femoral bone marrow cells from a representative animal (#21131) were split into CD38^+^ and CD38^−^ fractions (Fig. 3a) or into CD20^+^ and CD20^−^ fractions (Fig. 3b) by MACS and the frequency of measles-specific and tetanus-specific antibody-secreting cells were determined directly ex vivo by ELISPOT. These results indicate that measles-specific plasma cells outnumbered tetanus-specific plasma cells by about 5-to-1 among unfractionated bone marrow cells and although they could not be detected among CD38^-^ or CD20^+^ fractions, we observed a similar ratio of antigen-specific plasma cells among the CD38^+^ and CD20^-^ fractions, respectively. Together this indicates that similar to the long-lived plasma cells isolated from human bone marrow^38^, the long-lived antigen-specific plasma cells in rhesus macaques were comprised of CD38^+^CD20^−^ cells.

Bone marrow biopsies are typically drawn from the iliac crest and this represents the most accessible site for analyzing bone marrow-derived plasma cells in humans. In contrast, most studies on bone marrow-derived plasma cells in mice have been limited mainly to the femur due to the small size of the bones in this animal model. The adult human skeleton consists of 206 bones and it is unclear if antigen-specific plasma cells home preferentially to different locations or if they are equally distributed among different bone marrow compartments. Although it was not feasible to examine plasma cell frequencies among all bones in a rhesus macaque, we focused our analysis mainly on the large bones of the appendicular skeleton (humerus, ulna, radius, iliac crest, femur, tibia, and fibula) in addition to rib and vertebrate (Fig. 3). For the long bones, we also compared plasma cell frequencies between proximal, middle, and distal sites within each bone but we did not find any consistent differences between these various locations within the same bone marrow compartment. When measured directly ex vivo by ELISPOT at the time of necropsy, tetanus-specific antibody-secreting plasma cells were found at the highest frequency in the tibia, femur, and humerus (30, 21, and 19 ASC/10^7^ cells, respectively) with little or no difference in tetanus-specific plasma cell frequencies among B cell-depleted experimental animals vs. untreated controls (Supplementary Fig. 2) as expected based on serum antibody levels (Figs. 1 and 2). The ELISPOT data from all 6 animals was averaged (Fig. 3) and when compared to the femur, there were significantly fewer tetanus-specific plasma cells identified in the radius, rib, vertebrate, or iliac crest (5.5, 2.4, 2.1, and 3.0 ASC/10^7^ cells, respectively; *P* < 0.05, mixed effect negative binomial regression, Dunnett adjusted). We also found a non-significant trend towards lower numbers of tetanus-specific plasma cells in the fibula (6.8 ASC/10^7^ cells) and ulna (10.0 ASC/10^7^ cells) compared to femur (Fig. 3c). One explanation for the reduced frequency of tetanus-specific plasma cells in these particular bone marrow compartments could be that they have fewer total IgG-secreting plasma cells in general. We examined this issue by measuring the frequency of total IgG-secreting cells by ELISPOT in parallel to the tetanus-specific ELISPOT experiments (Fig. 3d). Femoral IgG-secreting plasma cells were found at a frequency of ~ 12,600 ASC/10^6^ bone marrow cells and although the numbers were similar among humerus, ulna, radius, femur, and tibia, we found a trend towards fewer total IgG-secreting plasma cells in the fibula (*P* = 0.08, mixed effect negative binomial regression, Dunnett adjusted) and significantly fewer IgG-secreting cells in the rib, vertebrate and iliac crest when compared to the femur (*P* < 0.05, mixed effect negative binomial regression, Dunnett adjusted, Fig. 3d). However, the lower overall frequency of IgG-secreting cells in these bone marrow compartments does not fully explain the differential localization of vaccine-induced plasma cells in this study since significantly fewer tetanus-specific plasma cells were still observed in vertebrate and iliac crest even after normalizing for the number of total IgG-secreting plasma cells at each of these sites (Fig. 3e, *P* < 0.05, rank based mixed effect linear regression, Dunnett adjusted). Likewise, the normalized number of tetanus-specific/total IgG-secreting ASC was lower in radius and rib when compared to femur despite not reaching statistical significance (*P* = 0.13 and *P* = 0.06, respectively, rank based mixed effect linear regression, Dunnett adjusted). These results indicate that following tetanus vaccination in early adolescence, there were significant differences in the frequency of vaccine-induced antigen-specific plasma cells found in different bone marrow compartments when examined 10 years after immunization. This was an unexpected finding and leads to many intriguing questions regarding the potential mechanisms underlying differential localization or maintenance of plasma cells within these unique bone marrow sites.

### Identification of bromodeoxyuridine^+^ plasma cells

**Fig. 4:**
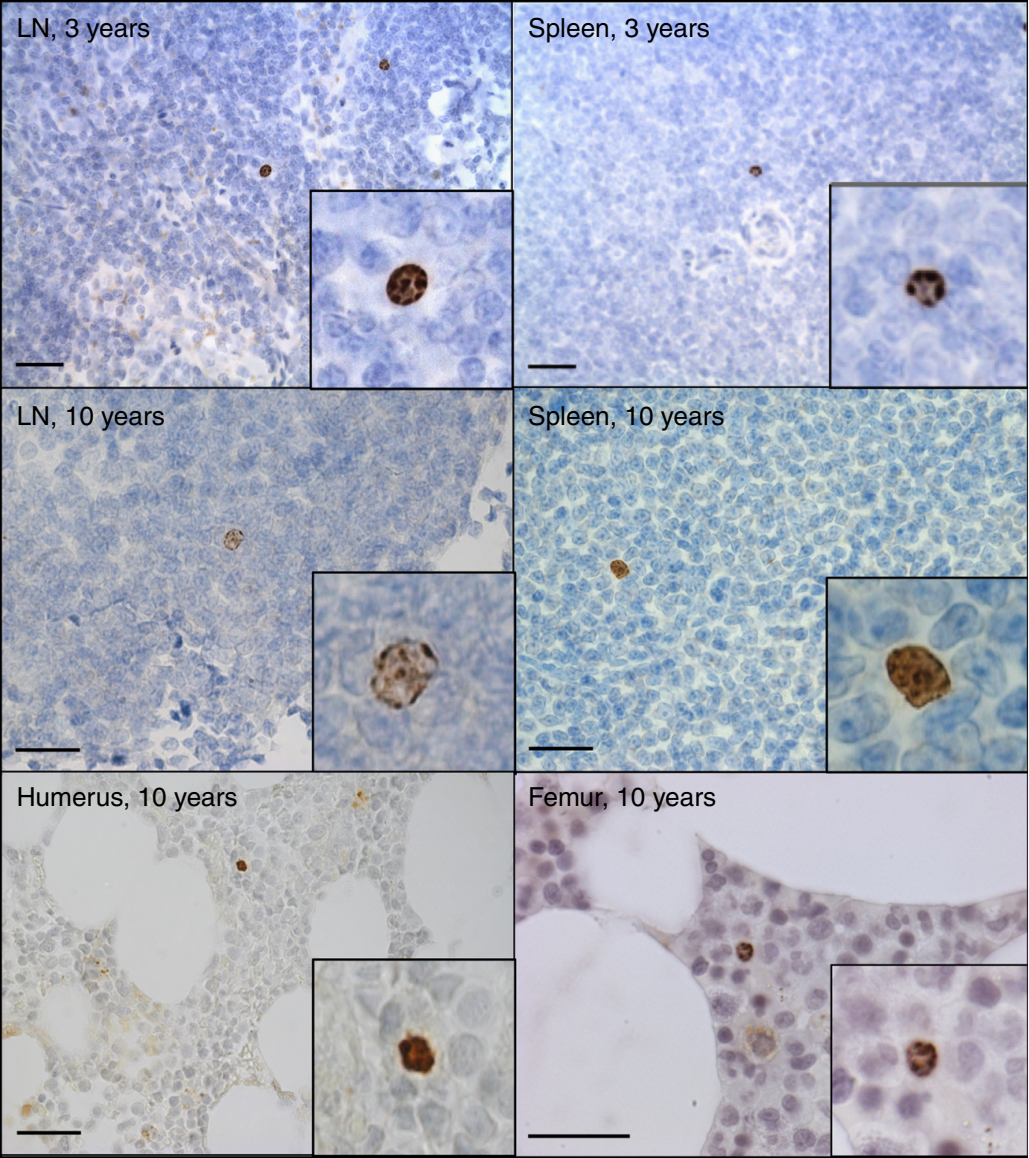
Identification of BrdU^+^ plasma cells at 3.5 and 10 years after BrdU administration. Each panel shows a representative microscopic image of tissue samples from rhesus macaques after immunohistochemical staining for BrdU^+^ cells. Paraffin-embedded bone marrow and tissue samples were stained for BrdU and counterstained with Mayer’s hematoxylin. BrdU^+^ plasma cells were identified based on their size and their characteristic “clock face” nuclei (see inset images). Sections showing draining lymph node (LN) or spleen at 3.5 years after primary vaccination were obtained by surgical removal from experimental CD20-depleted animals (Rh#21139 and Rh#21131, respectively). LN and spleen samples examined at 10 years post-vaccination were obtained from one of the control animals (Rh#20923) whereas sections of humerus and femur came from experimental CD20-depleted animals (Rh#21128 and Rh#21131, respectively) at necropsy. A 20 μm scale bar is included in each panel

Bromodeoxyuridine (BrdU) is a thymidine analog that can be administered to animals for a specified period of time during which it is incorporated into the DNA of dividing cells. If cells cease to divide, then the BrdU^+^ signal can be maintained indefinitely. Antibodies specific for BrdU can then be used to identify cells that proliferated at the time of BrdU administration and thus provide a useful approach for determining the survival of long-lived cells in the absence of further cell division. BrdU incorporation studies in mice demonstrated that IgG^+^ plasma cells could be identified by flow cytometry for up to 3 months after cessation of BrdU administration and indicated that, relative to the lifespan of a mouse, these represented a long-lived non-dividing cell population^7^. This is consistent with prior studies in rats that also identified long-lived cells with plasma cell morphology after administration of^3^H-thymidine^6,8^. However, in each of these examples there was still a remote possibility that plasma cells could be repopulated by memory B cells that differentiate into plasma cells with little or no proliferation and thereby retain the BrdU^+^ or^3^H-thymidine^+^ signal when examined at later time points. In our studies, rhesus macaques received BrdU for 12 days after the second, third, or fourth vaccination and we used immunohistology to identify BrdU^+^ cells at 3.5 or 10 years after vaccination (Fig. 4). As noted in Fig. 1, the spleen and draining lymph nodes were surgically removed ~3.5 years after primary vaccination at the time that peripheral anti-CD20 depletion was repeated. When we examined the spleen and lymph node samples from these animals histologically, we identified BrdU^+^ cells with characteristics of plasma cells including a larger size distribution compared to lymphocytes and a cartwheel or “clock face” chromatin pattern^42,43^ that becomes readily apparent when staining for BrdU^+^ DNA. This plasma cell morphology^42,43^ is consistent with the CD38^+^CD20^−^ plasma cell phenotype described in Fig. 3.

Since all four of the experimental memory B cell-depleted animals underwent splenectomy and lymph node excision, analysis of long-lived BrdU^+^ plasma cells in the spleen and lymph node at 10 years after vaccination was performed only among the intact control animals. Importantly, we also identified BrdU^+^ cells with plasma cell morphology in the bone marrow (e.g., humerus and femur) at 10 years after vaccination in animals that had previously undergone anti-CD20 treatment to remove memory B cells several years earlier (Fig. 4). The identification of BrdU^+^ cells with plasma cell morphology by histology provides independent confirmation of long-lived plasma cells that supports the functional data (Figs. 1 and 2) demonstrating that antibody-secreting plasma cells may survive for 10 years or more in the absence of repopulation by memory B cells.

## Discussion

Pre-existing antibodies often represent the first line of defense against microbial pathogens and are the basis for protective immunity elicited by many successful vaccines. Studies in rodents have shown that antibody-secreting plasma cells may survive for several weeks or months but this work is limited by the short lifespan of the host. In these current studies, we examined the mechanisms underlying long-term antibody maintenance in rhesus macaques, a species with a lifespan more similar to humans. We found that antibody responses following tetanus vaccination were long-lived and likely to provide lifelong protective immunity against this disease. Antibody responses to other viral and vaccine antigens were similarly long-lived regardless of memory B cell depletion by anti-CD20 administration and surgical removal of the spleen and draining lymph nodes. When we examined different bone marrow compartments, we were surprised to find that tetanus-specific plasma cells were not equally distributed among different bone marrow sites but instead were enriched in certain long bones such as the femur, tibia, and humerus. We used BrdU incorporation studies as an independent approach to identifying long-lived cells with plasma cell morphology in the bone marrow for ≥ 10 years after vaccination/BrdU administration. Altogether, these studies provide a framework in which the maintenance of long-term serum antibody responses appears to be maintained by long-lived plasma cells independently of memory B cells and indicates that vaccine approaches that elicit long-lived plasma cells will be the most effective at maintaining persistent antibody responses.

Potential mechanisms underlying the maintenance of long-term antibody responses can be generally divided into memory B cell-dependent and memory B cell-independent models^16^. The two most commonly asserted memory B cell-dependent models involve either persisting antigen or non-specific polyclonal activation of memory B cells to proliferate and differentiate into antibody-secreting plasma cells. As noted in Fig. 1, we found that antibody half-life estimates changed dramatically over the course of time and similar to other models^44^ it is likely that early antibody responses are determined by a combination of short-lived and long-lived plasma cells and the generation of new plasma cells in response to antigen depots. However, by 2–3 years after vaccination, antibody decay rates stabilize and there are several lines of evidence indicating that maintenance of long-term antibody responses at this stage of the immune response are no longer antigen-dependent. For example, studies involving the persistence of radiolabelled antigens revealed that > 99% of injected antigen is degraded within 2–4 days and the remaining antigen, presumably in the form of immune complexes, decays with about an 8-week half-life^16,45^. Addition of alum adjuvant is unlikely to greatly alter the duration of antigenic stimulation since intact alum granulomas may no longer be immunogenic after 14 days^46^. In our studies, we waited for over 1 year after the final vaccination in order to allow persisting antigen to dissipate and antibody titers to stabilize before performing memory B cell depletion by anti-CD20 administration (Fig. 1). We further removed the potential caveat of persisting antigen/immune complexes in lymphoid tissues by surgically removing the spleen and draining lymph nodes and repeating memory B cell depletion at 3.5 years after primary vaccination and we still found no significant impact on pre-existing antibody maintenance against tetanus (Fig. 1) or other virus and vaccine antigens (Fig. 2). A recent study identified long-lived plasma cells in the human intestine^47^ and it is possible that antigen-specific memory B cells reside in these locations as well. Although we did not examine intestinal sites of antibody production, we believe that it is unlikely that there are appreciable numbers of gut-associated tetanus-specific memory B cells in comparison to the spleen and draining lymph nodes after intramuscular DTaP vaccination. In addition, it may be unlikely that gut-associated memory B cells would be involved with repopulating IgG-secreting plasma cell populations in the bone marrow without migrating through the bloodstream and producing more memory B cells in order to sustain both memory B cells and plasma cell numbers. Since circulating tetanus-specific memory B cells remain below detection after anti-CD20 depletion (Fig. 1d), this would suggest that memory B cells from tertiary sites besides the spleen and lymph nodes play little or no role in maintaining systemic antibody production.

In terms of a model of memory B cell-dependent humoral immunity based on polyclonal non-specific memory B cell activation^16–18^, we likewise found no sign of loss in antibody maintenance despite effective removal of memory B cells to below our limits of detection (Fig. 1d). According to the polyclonal stimulation model^16–18^, ongoing or intermittent infection is believed to activate antigen-specific memory B cells through T cell-mediated cytokines or TLR-based activation that in turn, would result in proliferation and differentiation into more antibody-secreting plasma cells. Although B cells are readily activated to proliferate non-specifically in vitro^17,48^, the in vivo relevance of these results is difficult to ascertain since subsequent studies have been unable to demonstrate non-specific bystander activation resulting in increased levels of unrelated antibodies despite close serological monitoring of human subjects after defined episodes of vaccination or infection^16,49,50^. Our data (Figs. 1 and 2) and B cell ablation studies in humans^51–58^ together indicate that long-term serum antibody responses can be maintained for prolonged periods of time without requiring polyclonal stimulation of memory B cells.

Consistent with our results and a model of memory B cell-independent antibody production by long-lived plasma cells, several studies in humans have shown that antibody responses to common virus and vaccine antigens are relatively stable when followed for 4 months to 2.5 years after anti-CD20 depletion^51–57^ or CD19-directed chimeric antigen receptor-based adoptive T cell therapy^58^. However, since rituximab may not efficiently deplete B cells from lymphoid tissues^25–31^, the potential role of memory B cells in maintaining long-term antibody responses in humans had remained unclear. Our results differ somewhat from our prior work performed in mice in which gamma-irradiation was used to eliminate memory B cells in vivo^9^. After irradiation, antibody responses persisted but declined in comparison to untreated controls. We believe that this may have been due to non-specific damage to plasma cells or their supportive microenvironment and that this led to shorter plasma cell survival curves. In our current studies in Rhesus macaques, we used targeted depletion of CD20^+^ B cells instead of whole-body irradiation and this may explain why we found no significant difference in antibody titers between memory B cell-depleted animals and control animals (Fig. 1, *P* = 0.80, Mann-Whitney test). Tetanus-specific plasma cells in the spleen or draining lymph nodes were rare, residing either near or below our limits of detection by ELISPOT at 3 or 10 years after vaccination (3.6 per 10^7^ spleen cells or ≤ 1 per 8 × 10^6^ lymph node cells). This, along with the continued maintenance of serum antibody titers despite the surgical removal of the entire spleen and draining lymph nodes (Figs. 1 and 2), provides further supportive evidence indicating that the bone marrow is indeed a major site of systemic vaccine-induced antibody production in non-human primates.

ELISPOT analysis of tetanus-specific plasma cells revealed that 10 years after immunization, long-lived vaccine-induced plasma cells were preferentially identified in certain bone marrow compartments (e.g., femur, tibia, humerus) in contrast to other bone marrow sites (e.g., rib, radius, vertebrae, iliac crest) (Fig. 3). This was an unexpected finding and leads to several questions regarding the nature of these results. Are these observed differences due to the site of vaccination, the type of antigen, the age at vaccination, changes in bone marrow composition over time (e.g., hematopoiesis vs. adipose deposits) or other currently unknown criteria that influence the preferential homing or survival of plasma cells? Could preferential localization to specific bone marrow sites be determined by the age at the time of vaccination or the age at time of in vivo analysis? Rhesus macaques mature more rapidly than humans and reach reproductive maturity by 3–5 years of age and have an average lifespan of about 27–35 years in captivity^37^. The animals in our study were ~ 3 years old at the time of vaccination and bone marrow analysis was performed at ~ 14 years of age. Newborn mammals initially have no fat in their bone marrow but during the aging process, fat accumulates in the bone marrow and reaches ~70% of the marrow space in appendicular bones by adulthood in humans^59^. Similar to humans, we found that long bones in adult rhesus macaques (femur, humerus, etc.) contained substantial numbers of adipocytes and the BrdU^+^ cells with plasma cell morphology were often surrounded by adipocytes (Fig. 4). It is unclear if the fat content of different bone marrow sites has an impact on the localization or survival of plasma cells in bone marrow or if other cell types/survival factors may play a larger role with plasma cell maintenance^60–66^. A time course study following vaccination will likely be necessary to determine if particular bone marrow compartments are preferentially seeded with newly generated plasma cells. Alternatively, it is possible that all bone marrow sites initially acquire a similar frequency of vaccine-induced plasma cells but some sites may be more capable of sustaining plasma cell numbers long-term in comparison with others. Elucidating the factors underlying this new finding will be important for better understanding the bone marrow compartment and its contribution to maintaining long-term humoral immunity.

Together, our studies demonstrate that following depletion of peripheral CD20^+^ B cells (including tetanus-specific memory B cells) by anti-CD20 administration and surgical removal of the spleen and draining lymph nodes, prolonged antigen-specific serum antibody titers continued to be maintained for over 10 years after vaccination by long-lived plasma cells that reside in the bone marrow compartment. Further studies are needed to determine why vaccine-induced plasma cells are preferentially localized in certain bone marrow sites in contrast to others (Fig. 3) and a better understanding of the mechanisms involved with determining the lifespan of individual plasma cells will be critical for developing new and more effective vaccine approaches capable of eliciting long-term protective humoral immunity.

## Methods

### Rhesus macaques

The study was performed in strict accordance with the recommendations described in the Guide for the Care and Use of Laboratory Animals of the National Institute of Health, the Office of Animal Welfare and the United States Department of Agriculture. All animal work was approved by the Oregon National Primate Research Center Institutional Animal Care and Use committee. The ONPRC has been continuously accredited by the American Association for Accreditation of Laboratory Animal Care since 1974 (PHS/OLAW Animal welfare Assurance #A3304–01). The study included 4 experimental animals (anti-CD20 treatment, splenectomy, surgical removal of draining lymph nodes) and 2 control animals for comparison. Animals with a prior history of diarrhea were excluded from enrollment in the study. Rhesus macaques (*Macacca mulatta*) were housed in either small groups, paired, or individual housing during the course of the study and fed twice daily with a standard commercial primate chow with water available ad libitum. Blood draws, vaccinations, and BrdU administration were performed under ketamine or telazol anesthesia. For surgery, ketamine and isoflurane were used for anesthesia and all efforts were made to minimize pain. Animals (males, Indian-Chinese origin, 2.8–3.2 years of age) were vaccinated four times at monthly intervals with Tripedia (Diphtheria and Tetanus Toxoids and Acellular Pertussis Vaccine Adsorbed; DTaP), by intramuscular injection into the quadriceps muscle. BrdU (25 mg/kg) was dissolved in sterile PBS and administered intravenously for 12 consecutive days starting four days after the second (Rh#20923 and Rh#21169), third (Rh#21091 and Rh#21139), or fourth (Rh#21128 and Rh#21131) vaccination (2 animals/group).

### B cell depletion

CD20^+^ B cells were depleted from four animals by administering anti-CD20 antibody (Rituximab; 20 mg/kg, Genentech) three times at 1-week intervals. Each of the vaccinated rhesus macaques had similar antibody responses to tetanus and there were no selection criteria used to determine the non-randomized group allocation with the exception of standard animal husbandry considerations involving feasibility of group or paired housing. The first 3-dose depletion regimen was performed 14 months after the fourth vaccination and the second 3-dose depletion series was performed at 3.5 years after the fourth vaccination, which was ~3 months after recovery from splenectomy and surgical removal of draining lymph nodes. Analysis of the efficiency of in vivo B cell depletion and immune reconstitution was performed by staining for residual CD22^+^ B cells (anti-CD22 antibody; 0.5 μl, Cat# MHCD2204, clone RFB-4) in PBMC at the indicated time points.

### Flow cytometry

PBMC were stained with α-CD20 (2.5 μl, Beckman Coulter, Clone B9E9), α-IgD (1 μl, Southern Biotech, Goat polyclonal antibody, δ heavy chain-specific, Cat#2030–09) and LIVE/DEAD® fixable Aqua dead cell stain (1:500 dilution, Life technologies, Cat#L34957). Only limited numbers of PBMC were available from Rh#21131 at early time points after memory B cell depletion and these samples were lost for technical reasons. To enumerate tetanus toxoid (TT)-specific memory B cells, 10 × 10^6^ cells were stained with 0.25 µg of rTT.C-FITC (List Biological Laboratories, Cat#196 A) and 0.1 μg of rTT.C-biotin. Specificity control samples were incubated with 0.25 µg of rTT.C-FITC (List Biological Laboratories, Cat#196) and 0.1 µg of biotinylated human serum albumin (HSA-biotin). HSA-biotin and rTT.C-biotin were prepared using EZ-Link™ Sulfo-NHS-LC-Biotin (Thermo Scientific Cat#21335) following manufacturer’s instructions. Cells were stained in 50 µL volumes overnight at 4 °C, washed, and incubated with streptavidin-APC (diluted 1:500, Cat#S868, Molecular Probes) for 30 min at 4 °C. Cells were washed again and fixed with 2% formaldehyde in PBS. Events were acquired on an LSR Fortessa (BD Biosciences) and analyzed with FlowJo software (FlowJo LLC).

### Histology

For histological analysis of plasma cells, paraffin embedded, formalin (10% buffered in phosphate) fixed tissues were cut in 5 µm slices and mounted on slides. Sections were stained using a modified protocol with a BrdU in-situ detection staining kit (Cat#550803, BD Pharmingen). Sections were deparaffinized in Xylene, rehydrated in ethanol (100%, 95%, 85%, 75, and 50%) before antigen retrieval in 10 mM citrate buffer, pH 5 in a pressure cooker. When the pressure cooker had a substantial amount of steam coming out the stopper, the slides were incubated for 10 min. The pressure cooker was then removed from the hot plate and left to cool for 1 h before opening and then the container with the slides was removed and allowed to cool slowly. The slides were dipped in water and then washed 3 × 3 min in PBS. Endogenous peroxidase was blocked with 3% H_2_O_2_ in methanol for 30 min and after a wash in water, the sections were incubated in 2 N HCl for 27 minutes. After 3 × 3 min washing steps in water followed by 3 × 3 min washing steps in PBS, the sections were pre-blocked with dilution buffer for 20 min before being incubated overnight in a humid chamber at 4 °C with anti-BrdU antibody (from the BrdU in-situ detection staining kit, Cat#550803, BD Pharmingen) on an orbital shaker. The following day, the sections were washed 3 × 3 min/each in PBS, incubated with Streptavidin-HRP for 30 min at room temperature on an orbital shaker, washed again and then the stain was developed using DAB substrate according to the manufacturer’s instructions. The sections were counterstained using Mayer’s hematoxylin, rinsed with water, dipped in 0.1% sodium bicarbonate a few times until they appeared blue and then dehydrated (50%, 75%, 85%, 95%, and 100% ethanol), cleared in xylene, and mounted with Permount.

### Bone marrow fractionation by MACS

Cryopreserved bone marrow cells were thawed and cell numbers were determined. Unfractionated cells were reserved for pre-fractionation ELISPOT and flow cytometry assays. Depletion of CD20^+^ cells was achieved by treating the BM cells with anti-human CD20 antibody-coated magnetic activated cell sorter (MACS) microbeads (25 μl beads for 50 × 10^6^ cells, Cat#130-091-105, Miltenyi Biotec) for 15 min at 4 °C followed by fractionation using a MACS LS column (Cat#130-042-401, Miltenyi Biotec) according to the manufacturer’s instructions. The CD20^-^ (unbound) flow-through fraction was collected and then the bound fraction containing CD20^+^ cells was eluted from the column. Both fractions were reserved for ELISPOT and flow cytometry analysis. CD38 fractionation was conducted by first labeling the bone marrow cells (Anti-human CD38; Caprico Biotechnologies Cat#100851) for 1 h at 4 °C prior to incubation with anti-biotin microbeads (100 μl beads for 50 × 10^6^ cells, Cat#130-090-485, Miltenyi biotech) for 15 minutes. The labeled and unlabeled cells were separated using the LS column as described for the CD20 bead-based fractionation. Cell numbers were determined for the enriched and the depleted fractions. The cell purity after CD38 fractionation was monitored by flow cytometry using anti-human CD38 antibody conjugated with Biotin (0.5 μg, Cat#100851, Caprico Biotechnologies) in combination with Streptavidin-APC (diluted 1:500, Cat#S868, Molecular Probes). Unfractionated and CD38-enriched samples were comprised of 16.1% and 26.8% CD38^+^ cells, respectively and the CD38-depleted fraction was comprised of 99.9% CD38^−^ cells. CD20 fractionation was monitored by flow cytometry using anti-human CD20 antibody conjugated with ECD (2.5 μl, Cat# IM3607U, Beckman Coulter). Unfractionated and CD20-enriched samples were comprised of 0.5% and 74.5% CD20^+^ cells, respectively and the CD20-depleted fraction was comprised of 99.9% CD20^−^ cells.

### ELISA and ELISPOT

Serum antibodies were measured using antigen-specific enzyme-linked immunosorbent assays (ELISA). Antigens included tetanus toxoid (0.125 μg/ml, Cat#582231, Calbiochem), diphtheria toxin (1 μg/ml, Cat#150, List Biological Laboratories), pertussis toxin (1 μg/ml, Cat#180 List Biological laboratories) filamentous hemagglutinin (0.5 μg/ml Cat #170, List Biological Laboratories), Pertactin (0.5 μg/ml BEI Resources, NR-34571), inactivated measles-Edmonston strain (1:250 dilution, AbD Serotec, PIP013), RhCMV strain 68-1 (1:1600 dilution, an in-house, detergent extract of RhCMV 68-1-infected rhesus fibroblasts treated with 2-mercaptoethanol to reduce non-specific binding to IgG) and H_2_O_2_-inactivated adenovirus (1:500 dilution, an in-house reagent consisting of Adenovirus serotype 5 with E1/E3 deleted). After coating with a previously optimized concentration of each antigen, the ELISA plates were washed and blocked with 5% nonfat dry milk for 1 h at room temperature. Serum samples were serially three-fold diluted and added to the plate and incubated for 1 h at room temperature. The plates were washed and incubated with horseradish peroxidase-conjugated goat anti-monkey IgG-Fc specific antibody (1:4000 dilution, Cat #GAMon/IgG(Fc)/PO, Nordic Immunology) for 1 h at room temperature. After washing the plates, colorimetric detection reagents containing 0.4 mg/ml *o*-phenylenediamine and 0.01% hydrogen peroxide in 0.05 M citrate buffer (pH 5) were added and the reaction was stopped after 20 min by the addition of 1 M HCl. Optical density at 490 nm was measured using a VersaMax ELISA plate reader (Molecular Devices). A standard (internal positive control) was included on all plates to normalize the ELISA values between plates and between assays performed on different days. Antibody titers were determined by log-log transformation of the linear portion of the curve using 0.1 optical density as the endpoint and performing conversion of the final values. Samples were tested in duplicate and paired samples with > 25% coefficient of variation (CV) were repeated. Tetanus-specific IgG titers from rhesus macaques were converted to international units/ml (IU/ml) after calibration with the human international serum standard, Tetanus Immunoglobulin TE-3, 120 IU/ml obtained from the National Institute for Biological Standards and Controls (Hertfordshire, England) using the polyclonal goat anti-monkey IgG-Fc detection reagent that was used for all rhesus macaque serum samples.

The frequency of tetanus-specific ASC, as well as total IgG-secreting ASC was measured by ELISPOT using plates coated with the same antigens used for ELISA. 96-well PVDF-bottomed plates (MAIPS- 4510, EMD Millipore) were coated with tetanus toxoid (1 μg/ml, Cat #582231, Calbiochem) inactivated measles (1:50 dilution, Cat #PIP013 BIO-RAD/AbD Serotec), or goat anti-monkey IgG/IgA/IgM antibody-heavy and light chain (10 μg/ml, Cat # 617-101-130, Rockland Inc.). The wells were blocked by incubating in RPMI medium containing 10% FBS for 1 h at room temperature. When feasible, ten wells were each loaded with one million cells/well for the tetanus-specific ELISPOT assay and serial three-fold dilutions of cells starting at 10^5^ cells/well were added to similar wells for anti-IgG ELISPOT. Plates were incubated at 37 °C for 6 h. After incubation, the plates were washed and HRP-conjugated goat anti-monkey IgG-Fc-specific (1:4000 dilution, Cat # GAMon/IgG(Fc)/PO, Nordic Immunology) was added. The plates were incubated overnight at 4 °C. Next, the plates were washed and the spots were detected by adding a filtered solution of 0.5 mg/ml of AEC (3-amino 9-ethyl carbazole) in 0.1 M sodium acetate containing 0.05% hydrogen peroxide. After gently washing the plates in running water, the detachable backings were taken off to facilitate rapid drying. The plates were air-dried for 6–24 h before counting the spots by visual inspection under a stereomicroscope.

### Splenectomy and inguinal lymph node removal

The spleen and both left and right inguinal (draining) lymph nodes were removed in the experimental group animals at ~3.2 years after the 4th vaccination. Positioning was in dorsal recumbency, with sterile prep and draping of the anterior abdomen. The abdomen was entered via 7 cm anterior ventral midline laparotomy, followed by placement of a medium Balfour and moistened lap sponges for visceral exposure. The spleen was placed in traction and the gastrosplenic ligament was transected. Moving from distal to proximal, the hilar splenic vessels and then the short gastric arteries were doubly ligated with either 3–0 coated Vicryl or large Hemoclips, and transected. The spleen was removed and after removal of the retractors, closure was accomplished with continuous 3–0 coated Vicryl in the rectus sheath and subcutis, followed by skin apposition with running intradermal 4–0 Monocryl. Prior to recovery, the inguinal region was prepped and draped bilaterally. Two cm skin incisions were created over the inguinal lymph nodes, followed by en bloc resection of all lymphatic tissue and surrounding adipose tissues using blunt dissection and the Bovie Closure was performed with continuous 4–0 Monocryl in the subcutis and skin. Recovery was on the operating room table until extubation. Medications used during surgery included Ketamine, oxygen, isoflurane, electrolytes, lactated ringer, Ophthalmic ointment, Bupivicain HCl, Lidocaine 1% with epinephrine, Glycopyrrolate, and Hydromorphone.

### Statistical analysis

Based on the observed antibody decay rate kinetics, the group sizes of *n* = 4 experimental animals and *n* = 2 controls, we would be sufficiently powered (80%) to detect an effect size of 3.8 in antibody decay rates between groups using two-sided *t*-test with significance level of 0.05. The Effect size was defined as mean difference divided by standard deviation. Due to the nature of long-term follow-up over the course of >10 years, researchers were not blinded to group allocation. However, partial blinding was used during data analysis as the final datasets were independently reviewed and analyzed by two biostaticians (B.P. and L.G.).

A two-stage approach was used to estimate a global decay rate of antigen-specific serum antibodies. First, regression fits on the log ELISA units vs. time after peak ELISA units (days) for each animal were used to estimate half-life.

1 $${\mathrm{ln}}\left( y \right) = \beta _0 + \beta _1T$$

In this case, y is ELISA units, *β*_0_ is intercept, $\beta _1$ is slope, and *T* is time after peak ELISA units (days). Then the average slopes ($\bar \beta _1$) were calculated, and used as an estimated decay rate. Half-life was defined by

2 $$\hat T_{\frac{1}{2}} = \frac{{{\mathrm ln}\left( {\frac{1}{2}} \right)}}{{\bar \beta _1}}.$$

This is a similar procedure to linear mixed modeling, random intercept and slope model that was used to estimate global intercept and slope. Due to the convergence and number of parameter problems as well as some periods with smaller number of observations, we adopted a regression fit on individual subjects rather than a random intercept and slope model. If $\bar \beta _1  >0$, then a half-life could not be reached and those were considered to be infinity.

For ELISPOT experiments, the differential distribution of tetanus-specific antibody-secreting cells (ASC) and total IgG-secreting ASC was determined using a random intercept mixed effect negative binomial regression model to not only account for within subject correlation, but adjusting for overdispersion^67^ in Poisson counts followed by post hoc comparisons in which the femur was used as the reference location and all other bone marrow compartments were compared to femur. For determining differences between bone marrow sites vs. femur after normalizing for the proportion of tetanus-specific ASC among total IgG-secreting ASC, we performed a rank based random intercept mixed effect linear regression model followed by post hoc comparisons in which the femur was used as the reference location and all other bone marrow compartments were compared to femur. The comparative data is presented as Dunnett adjusted *P* values.

### Data availability

The data supporting the results of this study are available within the article and its Supplementary Information files, or are available from the corresponding author upon reasonable request.

## Electronic supplementary material

Supplementary Information
