# Supplementary material for: Plasma cell survival in the absence of B cell memory
Source: Nat Commun. 2017 Nov 24;8:1781. doi: 10.1038/s41467-017-01901-w (PMC5701209; doi:10.1038/s41467-017-01901-w)
Supplement: Supplementary file 1 — Supplementary Information [file 41467_2017_1901_MOESM1_ESM.pdf]

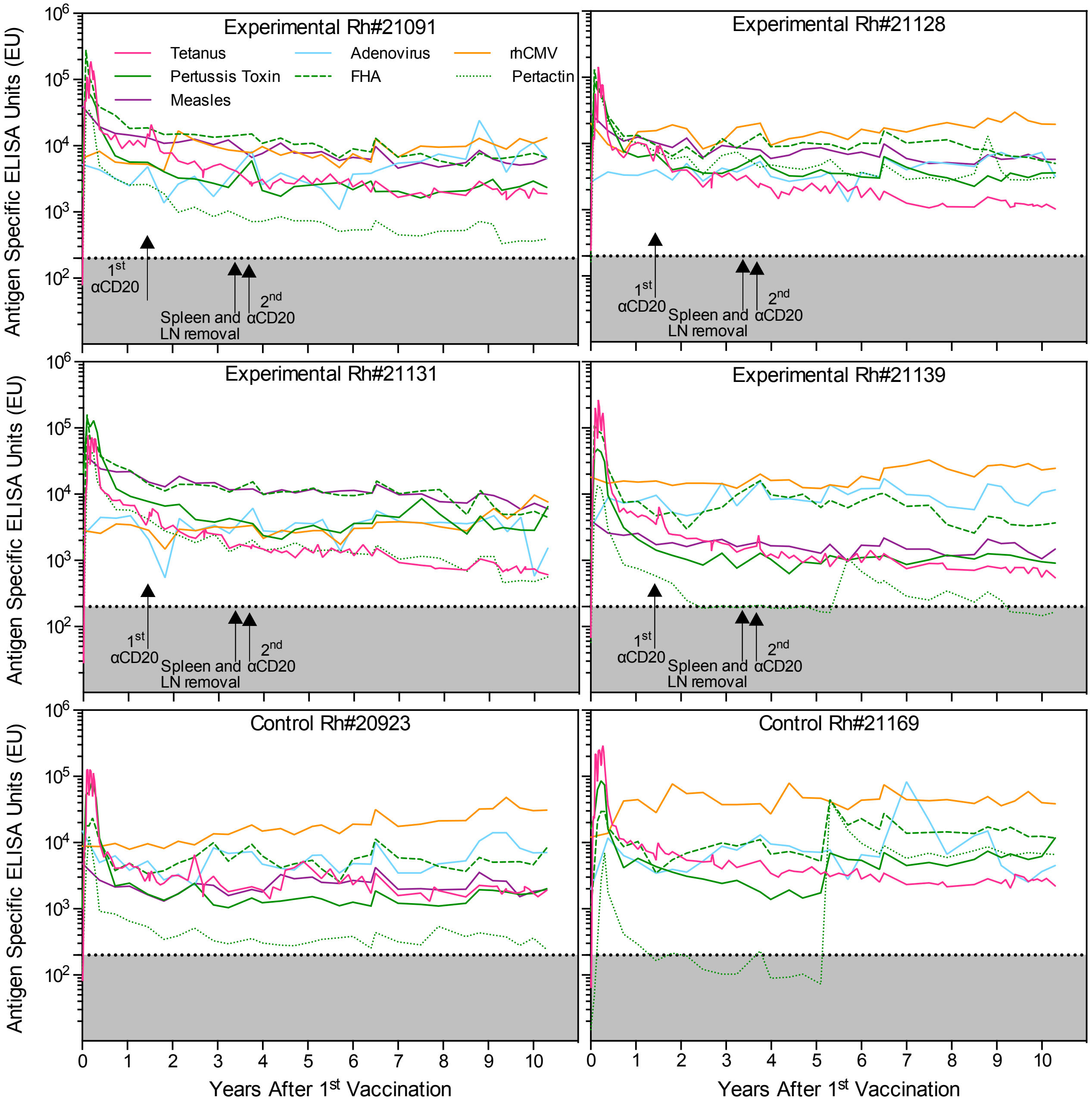

Supplementary Figure 1

Serum antibody titers were measured at the indicated time points for tetanus, pertussis toxin, pertactin, FHA (filamentous hemagglutinin), adenovirus, RhCMV (rhesus cytomegalovirus), and a paramyxovirus that is antigenically related to measles virus (Measles). Arrows indicate the dates when anti-CD20 administration was performed or when splenectomy and draining lymph nodes (LN) were surgically removed. Control animals, Rh#20923 and Rh#21169, did not have anti-CD20 treatment or surgeries performed. The gray shaded region (<200 ELISA units) represents the points in which ELISA titers become equivocal or are below the limit of detection. Control animal Rh#21169 was seronegative for measles virus antigen.

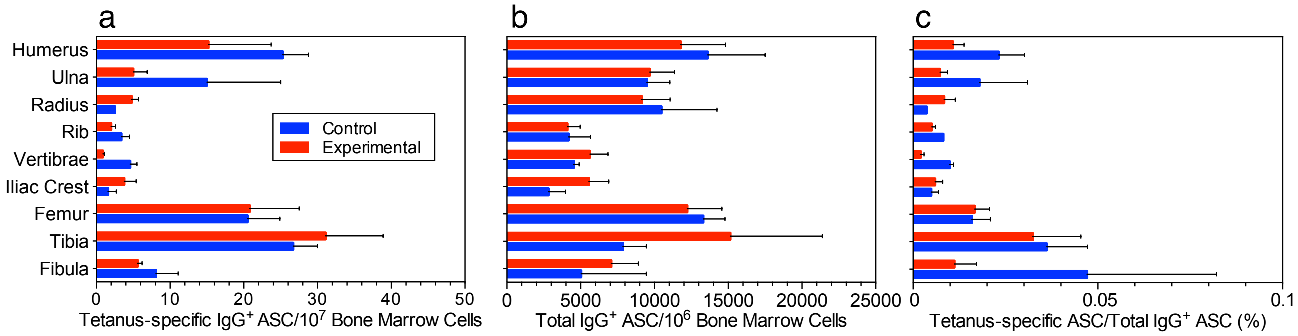

Supplementary Figure 2

Quantitation of tetanus-specific or total IgG-specific antibody-secreting cells (ASC) among experimental and control animals. The frequency of tetanus-specific ASC (a), the frequency of total IgG-secreting ASC (b) and the frequency of tetanus-specific ASC as a percentage of total IgG-secreting ASC (c) was determined by ELISPOT analysis for each of the indicated bone marrow sites (n = 4 experimental animals/group and 2 control animals/group). The bars represent the mean±S.E.M.
